# Neural oscillations promoting perceptual stability and perceptual memory during bistable perception

**DOI:** 10.1038/s41598-022-06570-4

**Published:** 2022-02-17

**Authors:** Michael Zhu, Richard Hardstone, Biyu J. He

**Affiliations:** 1 Neuroscience Institute, New York University School of Medicine, New York, NY 10016 USA; 2 Departments of Neurology, Neuroscience and Physiology, Radiology, New York University School of Medicine, New York, NY 10016 USA

**Keywords:** Consciousness, Perception, Object vision

## Abstract

Ambiguous images elicit bistable perception, wherein periods of momentary perceptual stability are interrupted by sudden perceptual switches. When intermittently presented, ambiguous images trigger a perceptual memory trace in the intervening blank periods. Understanding the neural bases of perceptual stability and perceptual memory during bistable perception may hold clues for explaining the apparent stability of visual experience in the natural world, where ambiguous and fleeting images are prevalent. Motivated by recent work showing the involvement of the right inferior frontal gyrus (rIFG) in bistable perception, we conducted a transcranial direct-current stimulation (tDCS) study with a double-blind, within-subject cross-over design to test a potential causal role of rIFG in these processes. Subjects viewed ambiguous images presented continuously or intermittently while under EEG recording. We did not find any significant tDCS effect on perceptual behavior. However, the fluctuations of oscillatory power in the alpha and beta bands predicted perceptual stability, with higher power corresponding to longer percept durations. In addition, higher alpha and beta power predicted enhanced perceptual memory during intermittent viewing. These results reveal a unified neurophysiological mechanism sustaining perceptual stability and perceptual memory when the visual system is faced with ambiguous input.

## Introduction

Visual input in the natural environment is often ambiguous due to shading, occlusion, clutter, and inherent complexity of natural objects^1^. Ambiguous images—wherein ambiguity of a visual image is sufficiently high as to trigger continuous perceptual alternations between possible interpretations—offer an excellent experimental paradigm to unravel how the visual system resolves perception when sensory input is ambiguous^2–4^. When viewing these ambiguous images, the visual input remains static while perceptual outcome changes spontaneously; this allows disentangling neural activities related to low-level sensory processing from those giving rise to the content of perceptual awareness^3,5^. Furthermore, this paradigm is well suited for investigating neural mechanisms that promote perceptual switching vs. perceptual stability, which manifest as spontaneous changes in perception and prolonged percept durations, respectively.

Studies in recent decades have focused on investigating neural activity related to perceptual switches. A large body of functional magnetic resonance imaging (fMRI) and brain stimulation studies of bistable perception (elicited by ambiguous images or binocular rivalry) revealed a right-lateralized frontoparietal network involved in perceptual switching (reviewed in^6^). Specifically, transcranial magnetic stimulation (TMS) of the superior parietal lobule (SPL) altered the rate of perceptual switching^7–9^, and fMRI studies revealed a brain network involving the SPL and lateral frontal regions that exhibit heightened activity around times of perceptual switches^10,11^, although the interpretation of the frontal activity remains controversial^6,12–14^.

While fMRI provides excellent spatial resolution, shedding light on neural mechanisms underlying perceptual stability requires monitoring brain activity with better temporal resolution using electrophysiology. Specifically, during the time periods between perceptual switches, the percept typically appears stable until the next switch event. Which neural processes are responsible for these brief periods of perceptual stability and how long they may last? Recent EEG studies suggest that the power and frequency of alpha oscillations may play a role in mediating perceptual stability^15,16^.

Interestingly, perceptual stability during bistable perception can be dramatically enhanced by using an intermittent presentation paradigm^17–21^. Here, instead of presenting a static ambiguous image, a blank period is inserted in between repeated presentations of the same image; this simple manipulation results in a significant decrease in perceptual switch rate—with some subjects experiencing no switches over several minutes^18,19^. Such perceptual stabilization is thought to result from perceptual memory, a form of memory that retains information about the most recent percept across the blank period^22^. The neural processes underlying perceptual memory remain unclear. We hypothesized that the same neural mechanisms promoting perceptual stability during continuous bistable perception might also support perceptual memory during intermittent presentation.

In this study, we asked three main questions: (1) Which neural mechanisms promote perceptual stability when viewing ambiguous images? (2) Do neural mechanisms promoting perceptual stability also support perceptual memory? (3) Does the ventral frontal cortex causally contribute to perceptual stability or perceptual memory?

To answer these questions, we used high-definition tDCS (HD-tDCS) to causally manipulate neural activity in two brain regions: right inferior frontal gyrus (rIFG) and occipital pole (Occ). tDCS was used because it noninvasively modulates spontaneous neural excitability with effects lasting more than two hours after stimulation offset, and HD-tDCS has been shown to have better spatial focality than conventional bipolar tDCS^23,24^. rIFG was chosen as the main target region because, among frontal regions, it is most consistently associated with perceptual switching^6^. In addition, the rIFG represents both the fluctuating perceptual content^25^ and prediction errors that slowly build up during a percept^26^. Occ was included to distinguish between potential effects of tDCS on frontal and visual regions, though we did not expect Occ stimulation to induce significant behavioral effects given a previous null finding using rTMS^27^. Using EEG data recorded from stimulation and sham conditions, we further investigated which neural activity supported perceptual stability and perceptual memory.

## Results

### Experimental design

**Figure 1:**
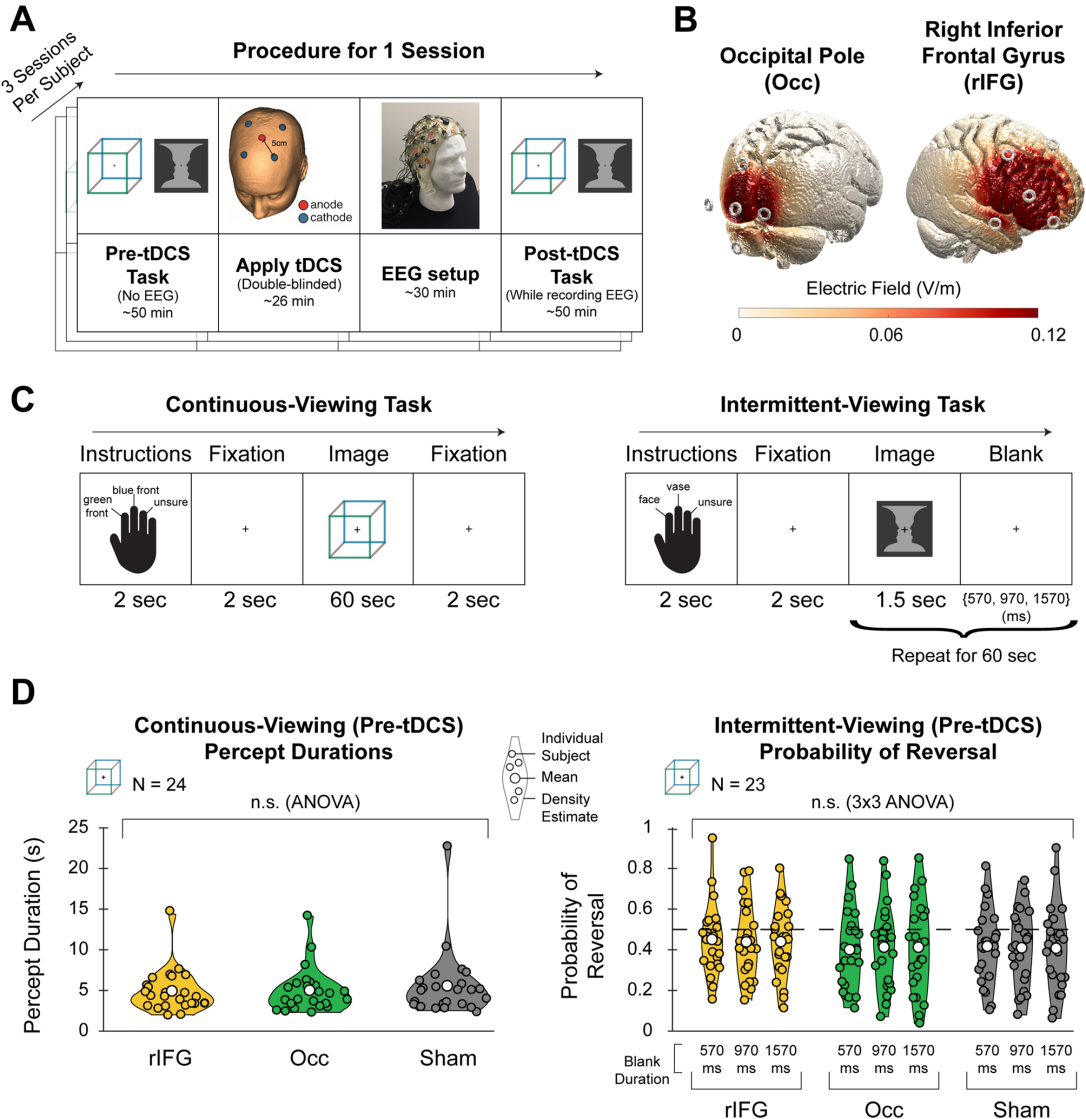
Experimental procedure and pre-tDCS behavioral results. (**A**) Each subject underwent the experiment on three different days. On each day, the experiment only differed in two possible ways: the region of tDCS stimulation and whether active or sham stimulation was applied. The experimental procedure on each day consisted of 4 steps: (1) Subject performed pre-tDCS task, (2) Double-blinded tDCS was applied over the target region, (3) tDCS cap was removed and the EEG cap was prepared, (4) Subject performed post-tDCS task under EEG recording, which used the exact same task structure as the pre-tDCS task. (**B**) The two target regions used, occipital pole (Occ) and right inferior frontal gyrus (rIFG), and estimates of the electric field over the cortex when applying 2.0 mA tDCS current; image generated using ROAST toolbox from^28^. (**C**) Subjects performed two task conditions: continuous-viewing (*Left*) and intermittent-viewing (*Right*). For both task conditions, half of the trials presented the Necker cube image and the other half presented the Rubin face-vase image. (**D**) Violin plots of behavioral data across subjects for cube trials in the pre-tDCS task (face-vase trials shown in Fig. S1A). Dotted line for intermittent-viewing data (*Right*) indicates 0.5 probability of reversal. The cube image was created by one of the authors (B.J.H.); the face-vase image was adapted from a figure in^29^.

To test whether perturbing neural activity in the rIFG causes changes in perceptual stability during bistable perception, we carried out an HD-tDCS experiment with a double-blind, within-subject cross-over design (N = 24). The experiment included two different ambiguous images, the Rubin face-vase illusion and the Necker cube (Fig. 1A). Each subject participated in three experimental sessions with adjacent sessions spaced > 3 days apart in order to ensure that aftereffects of tDCS from one session (lasting < 6 h,^24^) did not influence the next session.

The three experimental sessions differed in tDCS manipulation and included: (1) anodal HD-tDCS over the right inferior frontal gyrus (rIFG; Fig. 1B, *Right*); (2) anodal HD-tDCS over the occipital pole (Occ; Fig. 1B, *Left*); (3) sham stimulation over either the rIFG or Occ (location pseudorandomized across subjects). The order of stimulation conditions was fully counterbalanced across 24 subjects, and the primary investigator (M.Z.) was blinded to the stimulation condition during data collection and primary data analyses. Stimulation sites for each subject were determined based on the individual subject’s anatomical MRI, using a Neuronavigation system.

In order to control for potential day-to-day variation in perceptual behavior, each subject performed the same bistable perception task twice on the same day: once at the beginning of the session (pre-tDCS task) and once at the end of the session (post-tDCS task). In between tasks, HD-tDCS was applied offline—while the subject sat idle in a chair and was not viewing any specific images (Fig. 1A). All subjects completed the post-tDCS task within 2 h after the tDCS stimulation ended (mean ± s.d., 79.5 ± 8.3 min). The pre-tDCS task was identical to the post-tDCS task, except that the subject performed the post-tDCS task under EEG recording. The task consisted of two different conditions: continuous viewing and intermittent viewing. In continuous-viewing trials, subjects viewed an ambiguous image for 60 s and pressed one of three buttons (e.g., green-fronted cube, blue-fronted cube, or ‘unsure’) each time their perception switched (Fig. 1C, *Left*). In intermittent-viewing trials, subjects were presented an ambiguous image for 1.5 s followed by a blank screen lasting 570 ms, 970 ms, or 1570 ms; this sequence repeated for 60 s (Fig. 1C, *Right*). Each time the image appeared on the screen, the subject pressed a button to indicate their percept.

Critically, each task condition (continuous or intermittent) in both the pre-tDCS and post-tDCS time periods included two different ambiguous images (cube and face-vase). This allowed us to analyze the data for each image separately to perform a within-study check on reproducibility and generalizability of the findings across specific image details.

### Behavioral results of Pre-tDCS task

Figure 1D *Left* shows the distribution of median percept durations across subjects for continuous-viewing cube trials of the pre-tDCS task (Fig. S1A*Left* shows data for face-vase trials). We first performed a control analysis to test whether percept durations differed across experiment sessions conducted on separate days. A one-way repeated-measures ANOVA revealed that percept durations in the pre-tDCS task did not differ between session types (rIFG, Occ, or sham) (face-vase: F_2, 44_ = 0.508, *p* = 0.605, cube: F_2, 46_ = 1.010, *p* = 0.349); this is expected as the pre-tDCS task is not influenced by tDCS effects. We further tested the within-subject reliability of percept durations across pre-tDCS sessions by calculating a one-way intraclass correlation (ICC)^30,31^. Percept durations were highly consistent across sessions within the same individual (face-vase: ICC = 0.79, F_22, 46_ = 12.06, *p* < 1.59e−12, cube: ICC = 0.83, F_23, 48_ = 15.14, *p* < 6.44e−15), consistent with earlier findings^32^.

For intermittent-viewing trials in the pre-tDCS task, we first tested for differences in reversal rate (switches/min) between conditions using a two-way (3 × 3) repeated-measures ANOVA with session type and blank duration as factors. There was a significant effect of blank duration for both face-vase and cube trials (face-vase: F_2, 42_ = 41.306, *p* = 1.21e−10, cube: F_2, 44_ = 72.673, *p* = 1.14e−14), and, as expected, no significant effect of session type in the pre-tDCS task. This result replicates an effect previously shown: reversal rate decreases as longer blank durations are used^17,19^ (see Fig. S1B). However, the reversal rate metric is influenced by a ceiling effect because reversals can only be reported during image presentation and not blank periods (therefore, longer blank periods may be trivially associated with lower reversal rates). To address this issue, we calculated another metric, “probability of reversal,” which quantifies the probability that the percept switches after a blank period (Fig. 1D *Right* and Fig. S1A*Right*; see “Methods”, “Behavioral measures”). Using the logit-transform of this probability metric as the dependent variable, we performed the same two-way (3 × 3) repeated-measures ANOVA and found a significant effect for blank duration in face-vase trials only, with longer blank durations associated with a higher probability of reversal (F_2, 42_ = 11.31, *p* = 1.18e−4; Fig. S1A, right).

Overall, data from the intermittent viewing condition reveal an effect of perceptual memory consistent with prior literature, evident in both lower reversal rates when compared to the continuous-viewing condition (Fig. S1B) and a < 0.5 probability of reversal for all blank durations used (17/18 comparisons, Fig. 1D, *Right* and Fig. S1A, *Right*). However, we did not observe a clear effect of blank duration on perceptual memory as previously postulated.

### Null effects of tDCS on perceptual stability and perceptual memory

To determine the effects of tDCS on perceptual stability during bistable perception, we compared data from the post-tDCS task between experimental sessions. First, we verified the effectiveness of sham stimulation: at the end of each experimental session, subjects completed a brief questionnaire, which asked them to guess if they received “active stimulation or placebo” during the session. Subjects were unable to distinguish active tDCS sessions from sham sessions (proportion test, z = 1.447, *p* = 0.148), suggesting that our sham protocol was an effective control.

**Figure 2:**
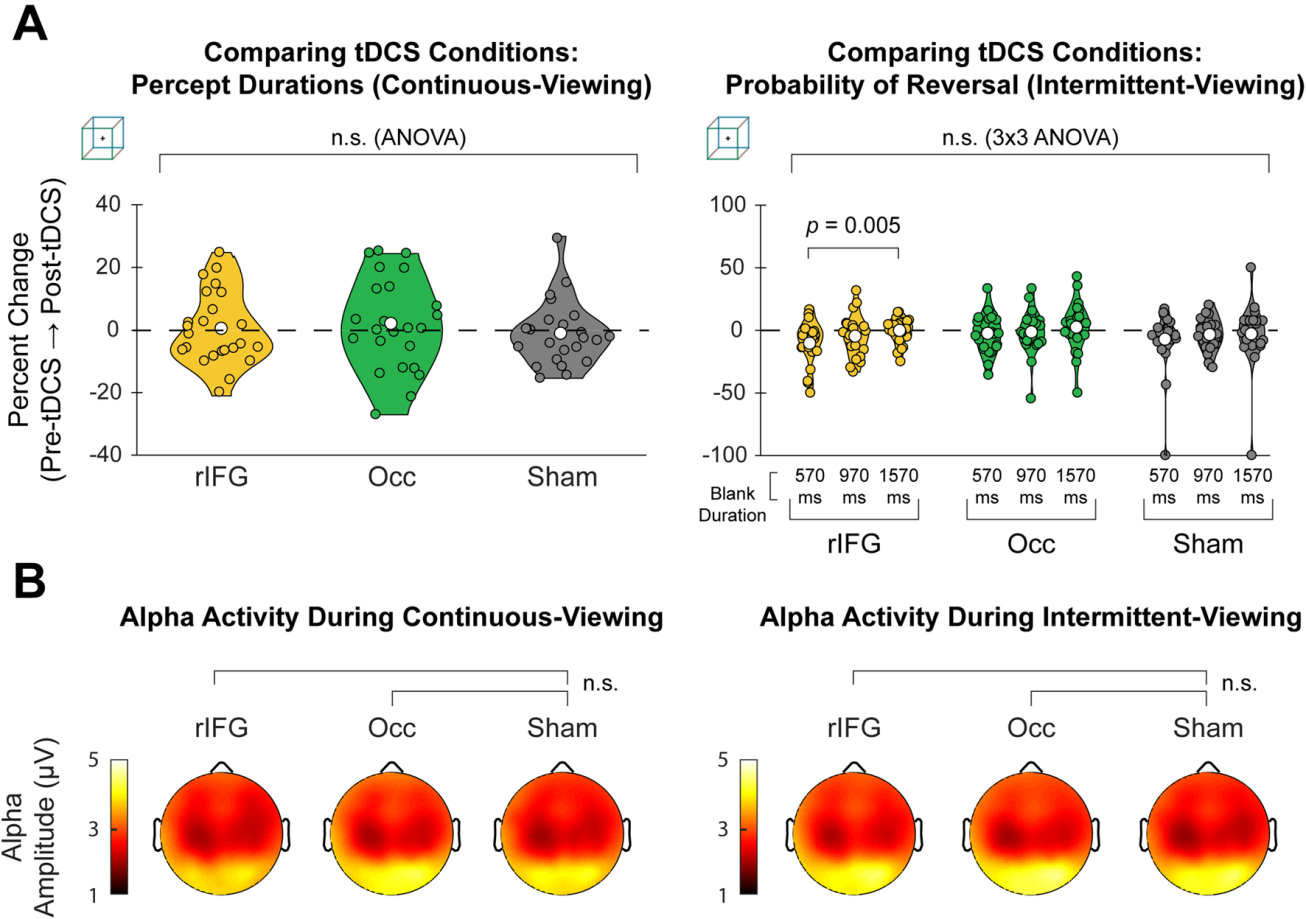
tDCS does not affect perceptual switching behavior or band-limited EEG amplitudes. (**A**) Violin plots show % changes in behavioral measures from pre-tDCS task to post-tDCS task for cube trials (face-vase trials shown in Fig. S2A). In the continuous-viewing condition (Left), percept durations did not significantly differ between tDCS conditions (repeated-measures ANOVA, F_2, 46_ = 0.391, *p* = 0.679). In the intermittent-viewing condition (Right), probability of reversal did not significantly differ between conditions (3 × 3 repeated-measures ANOVA, F_2, 44_ = 0.916, *p* = 0.386); post hoc tests showed that the only significant effect was between the shortest (570 ms) and longest (1570 ms) blank durations in the rIFG condition (t_22_ = −3.593, *p* = 0.005, Bonferroni-corrected). (**B**) Topo-plots show mean alpha amplitudes during continuous viewing (Left; *N* = 24) and during the 570 ms-blank condition of intermittent viewing (Right; *N* = 23) averaged across all subjects for cube trials (face-vase trials shown in Fig. S2B). Alpha amplitudes did not differ significantly between tDCS conditions (paired t-test, all *p* > 0.012, uncorrected across EEG channels). Similar null findings were obtained for other frequency bands (see Fig. S2B for face-vase trials).

We then proceeded to compare behavioral data between tDCS conditions. In order to control for potential day-to-day variability in perceptual behavior, we first calculated the percent change in median percept duration from the pre-tDCS task to post-tDCS task. Using change in percept duration (%) as the dependent variable, we performed a one-way repeated-measures ANOVA and did not find a significant effect of session type (i.e., tDCS condition) (Fig. 2A, *Left*, cube: F_2, 46_ = 0.391, *p* = 0.679; Fig. S2A, *Left*, face-vase: F_2, 44_ = 1.062, *p* = 0.354). Similarly, for the intermittent-viewing condition, we conducted a two-way (3 × 3) repeated-measures ANOVA with session type and blank duration as factors and % change in probability of reversal as the dependent variable; the only significant effect was blank duration for the cube trials (Fig. 2A, *Right;* F_2, 44_ = 3.513, *p* = 0.038). Post-hoc tests found that, only for the rIFG condition, the shortest blank duration (570 ms) resulted in significantly lower % change in probability of reversal than the longest (1570 ms) blank duration (t_22_ = -−3.593, *p* = 0.005, Bonferroni-corrected); however, this effect did not interact with session type (i.e. no difference between tDCS stimulation and sham). Session type did not have a significant main effect (face-vase: F_2, 42_ = 0.776, *p* = 0.467; cube: F_2, 44_ = 0.916, *p* = 0.386). Incorporating task timing (pre- vs. post-tDCS) as an additional independent factor in the ANOVAs (3 × 2 for continuous viewing, and 3 × 3 × 2 for intermittent viewing) also revealed no significant interaction effect on percept duration (in the continuous-viewing condition) or the probability of reversal (in the intermittent-viewing condition) (all *p* > 0.28).

Together, these results suggest that anodal tDCS over rIFG or Occ did not have a significant effect on perceptual switching behavior in either the continuous-viewing or the intermittent-viewing condition.

### Neural activity does not differ between tDCS and sham sessions

To determine the effects of tDCS on neural activity during the task, we analyzed band-limited amplitude in five canonical frequency bands: delta (1–4 Hz), theta (4–8 Hz), alpha (8–13 Hz), beta (13–30 Hz), and gamma (30–55 Hz). Specifically, we tested if the band-limited amplitude averaged across both percepts in the continuous-viewing condition or across blank periods in the intermittent-viewing condition differed between sham and active stimulation sessions. Paired t-tests between active tDCS (over rIFG or Occ) and sham sessions showed no significant differences at any EEG channel (all *p* > 0.012, uncorrected across EEG channels; see Fig. 2B for alpha-band results in cube trials, and Fig. S2B for results from all frequency bands in face-vase trials). Together with the null effects on behavior, these results suggest that anodal tDCS over rIFG or Occ did not have any significant effects on perceptual switching behavior or band-limited amplitude in our paradigm. However, as we did not record EEG in the pre-tDCS task, it remains possible that tDCS did cause reliable changes in neural activity, but these changes were small compared to the variability in neural activity across experimental days (since different tDCS conditions had to be carried out on separate days to ensure that aftereffects of one session do not influence a different session). Given this null finding, in the following analyses aimed at shedding light on the neural mechanisms underlying perceptual stability and perceptual memory, we combined EEG data from the post-tDCS task across the three sessions.

### Continuous viewing: alpha and beta amplitudes correlate with percept duration

**Figure 3:**
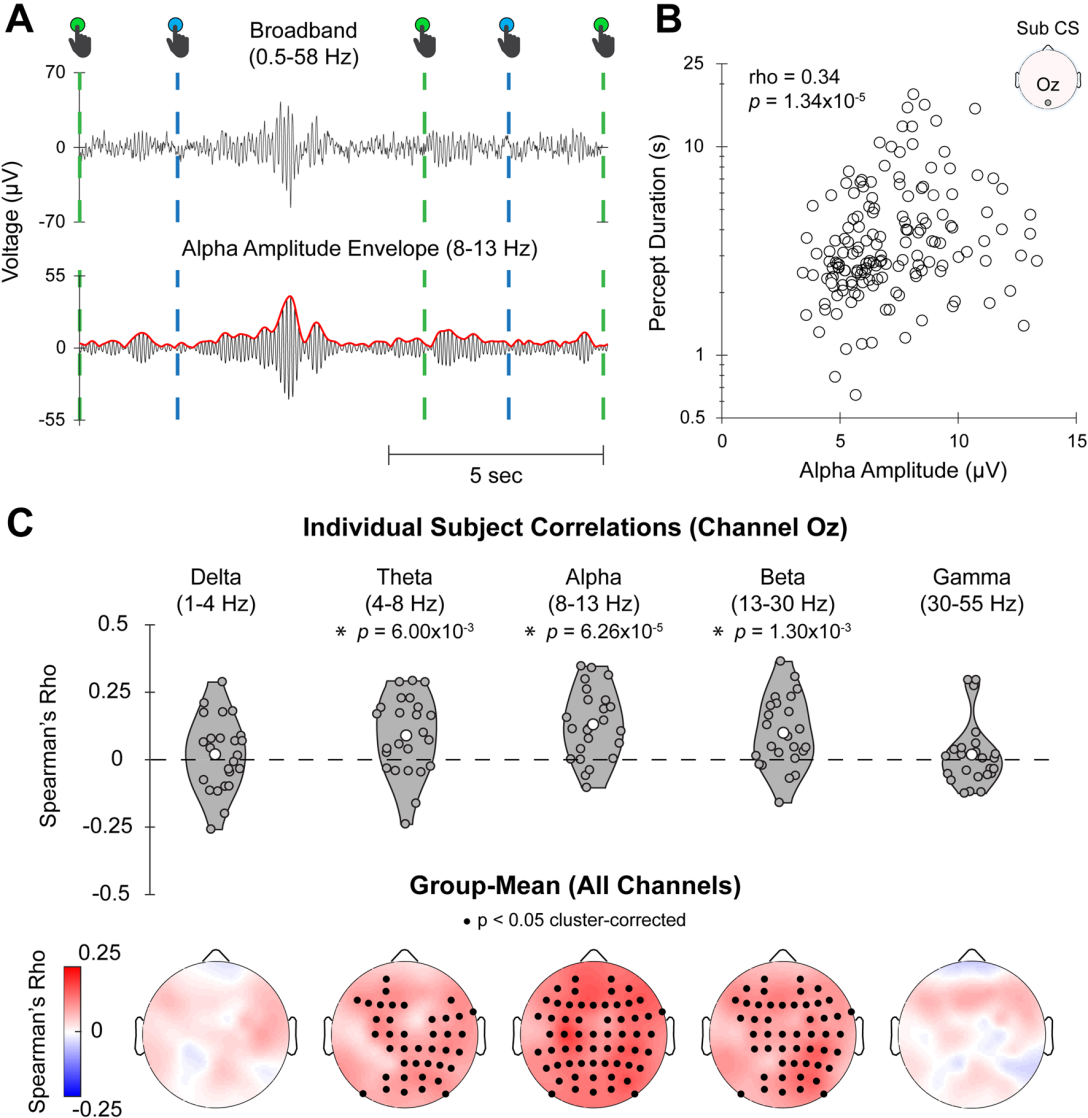
Longer percept durations correlate with higher alpha and beta amplitudes. (**A**) An example EEG recording over 12 s of continuously viewing the Necker cube. The signal was bandpass filtered in the alpha band (8–13 Hz) (black curve) and then Hilbert-transformed (red curve is the amplitude envelope). Dashed lines indicate times of button presses and color indicates whether the green-fronted cube or blue-fronted cube is reported as perceived. (**B**) Scatter plot shows all percept durations (*n* = 157) and the mean alpha amplitude during each percept (from channel Oz) for a single subject, from cube trials in the continuous-viewing condition. (**C**) *Top*: Violin plots show the distribution of Spearman rho values (calculated between EEG amplitude and percept duration over all percepts) across subjects for channel Oz (*p < 0.05, one-sample t-test on Fisher-z-transformed rho values, df = 23). *Bottom*: Topo-plots show group-averaged Spearman rho values. Black dots indicate channels with significant Spearman correlation (one-sample t-test on Fisher-z-transformed rho values, *N* = 24, corrected using cluster-based permutation test and thresholded at *p* < 0.05, two-tailed). Data for cube trials are shown here (see Fig. S3 for face-vase trials).

To identify neural activity that influences the stability of percepts, we tested whether EEG signal amplitudes in each frequency band were predictive of percept duration on a trial-to-trial basis during the continuous-viewing condition. We first segmented EEG data for each subject into trials, defined as the period of time from one button press to the next (excluding “unsure” button presses), and calculated mean band-limited amplitudes for each trial (Fig. 3A). Next, we computed Spearman correlation between percept duration and band-specific amplitudes across trials for each subject (see Fig. 3B for an example single-subject result from channel Oz for the alpha band). The distribution of Spearman rho values across subjects for each frequency band recorded from channel Oz is shown in Fig. 3C, *Top*. A group-level analysis was then calculated for each frequency band (see “Methods”, “Correlating band-limited amplitude with behavioral metrics”).

We observed widespread positive correlations between percept duration and EEG amplitude in the theta, alpha, and beta bands, with the strongest effect in the alpha band (*p* < 0.05, two-tailed cluster-based permutation test, N = 24) (cube: Fig. 3C, *Bottom*). The face-vase data showed qualitatively similar results, albeit with the theta band showing smaller significant clusters (Fig. S3). Overall, these results suggest that alpha (and, to a lesser extent, beta and theta) power significantly predicts percept duration on a trial-to-trial basis during bistable perception triggered by ambiguous images, and that this effect is reproducible and generalizable across multiple ambiguous images. Therefore, alpha- and beta-band activity appears to be involved in promoting perceptual stability.

### Intermittent viewing: alpha and beta amplitudes predict perceptual memory

**Figure 4:**
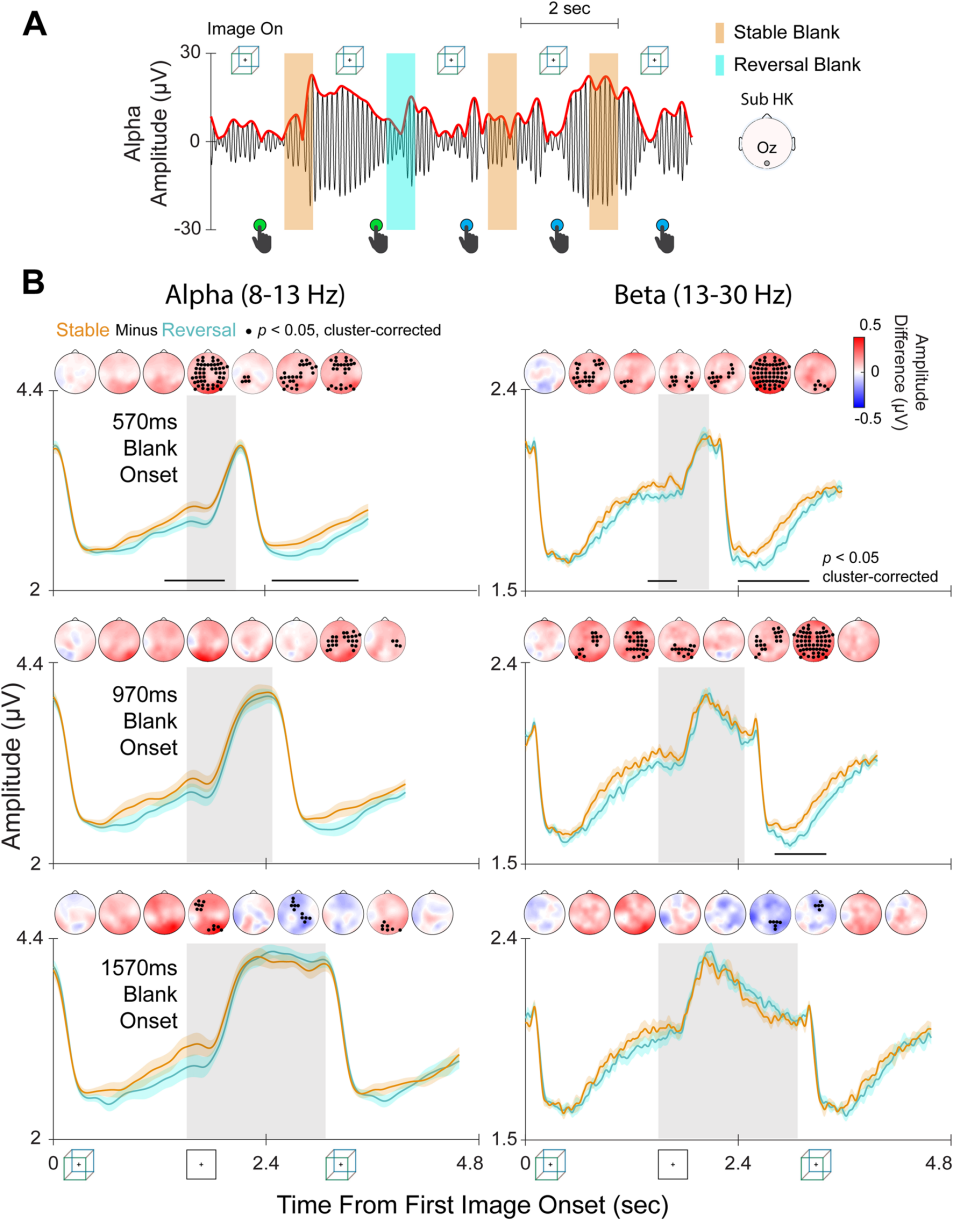
Alpha and beta amplitudes are higher in stable trials than in reversal trials. (**A**) An example EEG recording over 10 s of intermittently viewing the Necker cube (black curve is signal filtered in the alpha band and red curve is its amplitude envelope). Colored areas indicate blank periods; white areas indicate image presentation periods (1.5 s each) Green and blue button press symbols indicate reported perceptual switches to perceiving a green-fronted or blue-fronted cube, respectively. Each blank period was labeled a stable or reversal trial based on whether percepts before and after the blank period were the same or different. (**B**) Curves show alpha (*Left*) and beta (*Right*) amplitudes averaged across all channels for stable blank periods (orange curves) and reversal blank periods (cyan curves) (shading shows fixed-effects s.e.m.). Gray shaded areas indicate blank periods; white areas indicate image presentation periods (1.5 s each). Horizontal black bars indicate time periods of significant difference between stable and reversal trials (cluster-based permutation test, *p* < 0.05, two-tailed). Topo-plots show differences in band-limited amplitude (stable minus reversal trials) using 500 ms bins. Black dots indicate channels with significant differences between stable and reversal trials (cluster-based permutation test, *N* = 23, *p* < 0.05, two-tailed). Only data for cube trials are shown here (see Fig. S4 for face-vase trials).

We next investigated whether similar neural mechanisms also contribute to perceptual memory in the intermittent-viewing condition. To this end, we split all blank periods into two groups: those across which the percept was stable (stable blanks) and those after which the percept switched (reversal blanks) (Fig. 4A). We compared the time courses of band-limited amplitudes between these two groups, with each trial consisting of one blank period (‘stable’ or ‘reversal’) and the preceding and following image presentation periods. Figure 4B plots the time courses for alpha and beta amplitudes averaged across all EEG channels for ‘stable’ and ‘reversal’ trials for the cube image (for similar results for face-vase image, see Fig. S4). In the condition with the shortest blank duration (570 ms), activity in both the alpha and beta bands was higher for stable than reversal trials. This effect was significant (*p* < 0.05, cluster-based permutation test, two-tailed) during two different time intervals: the first spanning the end of the preceding image presentation to the beginning of the blank period (alpha: 1261–1944 ms; beta: 1379–1707 ms), and the second during the post-blank image presentation (alpha: 2478–3462 ms; beta: 2397–3201 ms) (Fig. 4B *First Row*). In the intermediate blank-duration (970 ms) condition (Fig. 4B *Second Row*), beta amplitude was also higher in stable than reversal trials during the post-blank image presentation (2811–3394 ms). In summary, averaged across all channels, alpha and beta amplitudes were higher in stable than reversal trials in the 570-ms and 970-ms blank conditions. Analysis of remaining frequency bands (delta, theta, gamma) did not reveal significant results (Fig. S5).

To determine which electrodes exhibited these effects, we subtracted activity in reversal trials from that in stable trials and averaged across 500 ms time bins for each electrode. Significant differences between stable and reversal trials were found for both the alpha and beta bands in all three blank-duration conditions (Figs. 4B and S4, black dots in topo-plots; cluster-based permutation test, N = 23, *p* < 0.05, two-tailed). Together, these results show that alpha and beta amplitudes are higher when the same percept is maintained across a blank period than when the percept switches, suggesting that higher alpha and beta amplitudes predict the existence of perceptual memory.

### Intermittent viewing: alpha and beta amplitudes correlate with the persistence of perceptual memory

**Figure 5:**
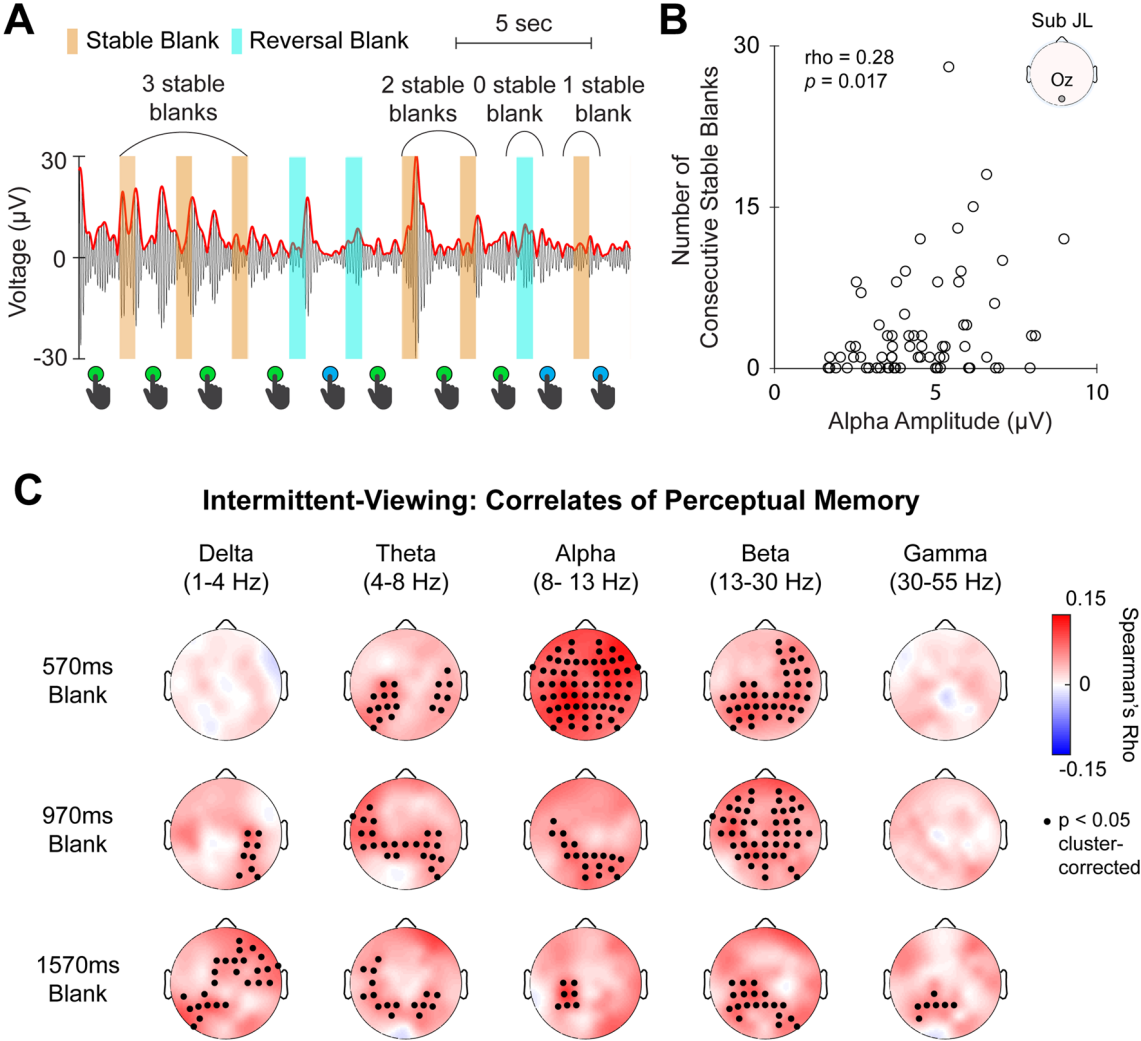
Alpha and beta amplitudes correlate with the persistence of perceptual memory. (**A**) The persistence of perceptual memory was assessed by the number of consecutive stable blank periods. The plot shows an example EEG recording over 20 s of an intermittent-viewing trial (black curve is signal filtered in the alpha band and red curve is its amplitude envelope) with its corresponding sets of consecutive stable blank periods. Stable blank periods are shaded in orange and reversal blank periods shaded in cyan. (**B**) Scatter plot shows all sets of consecutive stable blank periods and corresponding alpha amplitudes from channel Oz, for the cube trials from a single subject. (**C**) Spearman correlation was performed on each channel for each subject between the number of consecutive stable blanks and band-limited amplitude during the corresponding blank periods. Topo-plots show group-averaged Spearman rho values. Black dots indicate channels with significant Spearman correlation (cluster-based permutation test using Fisher-z-transformed rho values, *N* = 23, *p* < 0.05, two-tailed). Only data for cube trials are shown here (see Fig. S6 for face-vase trials).

Lastly, we tested whether alpha and beta activity contributes to the persistence of perceptual memory. To quantify the persistence of perceptual memory, we evaluated the number of consecutive blank periods across which perception remains the same (Fig. 5A). This metric is an integer value in the range of 0–N (where N equals the total number of blank periods in the trial). We used Spearman correlation to assess the relationship between this perceptual-memory metric and band-limited amplitudes averaged across the corresponding blank periods (see Fig. 5B for a single-subject example from channel Oz). Again, alpha and beta bands showed the most widespread significant clusters of electrodes (Fig. 5C; cluster-based permutation test, N = 23, *p* < 0.05, two-tailed). Smaller clusters were also found in the other frequency bands (delta, theta, and gamma), but the alpha and beta effects were the most consistent between image types (see Fig. S6 for results using face-vase trials). These results suggest that alpha and beta activity also contributes to the persistence of perceptual memory—that is, how long a percept stays stable across multiple interruptions in the sensory input.

In sum, our results reveal a unified set of neural mechanisms underlying perceptual stability (during continuous viewing) and perceptual memory (during intermittent viewing): the wax-and-wane of alpha and beta band power modulates both perceptual phenomena, contributing to the stability and persistence of a given perceptual experience, whether the visual input remains in view or temporally disappears from view.

## Discussion

We report evidence for the involvement of alpha and beta activity in promoting both perceptual stability and perceptual memory when the visual system is faced with ambiguity in sensory input. Specifically, higher alpha and beta power correlated with both prolonged percept durations during continuous viewing of ambiguous images and increased persistence of perceptual memory during intermittent viewing. This result reinforces previous studies showing that alpha oscillations play a role in mediating perceptual stability (using continuous viewing paradigms)^15,16^, and extends these previous findings by demonstrating a unified function of alpha and beta band activity in contributing to both perceptual stability and perceptual memory—two phenomena likely involved in generating the seamless perceptual experiences during natural vision, when sensory input is often ambiguous and fleeting. Lastly, contrary to our initial hypothesis, in a double-blind, sham-controlled study, anodal HD-tDCS over rIFG or Occ did not significantly influence perceptual switching behavior during this task. Below we discuss potential functional mechanisms of alpha and beta band activity, and the interpretation of the null tDCS finding.

### Possible functional roles of alpha (8–13 Hz) oscillations

Past studies have found links between alpha-band activity and perceptual stability during bistable perception^15,16^. In particular, we replicated the finding by Piantoni et al.^16^ showing that alpha power is positively correlated with percept duration across trials within an individual. Piantoni et al. interpreted this effect as reflecting a locking of the neurodynamical attractor state such that the state of neural activity supporting the current percept is stabilized. Although we agree that alpha oscillations seem to reflect stability of the current percept, the mechanisms through which this stability is achieved still remain unclear. Based on accumulating evidence in the past two decades, we instead interpret the correlation of alpha activity with percept durations as reflecting top-down feedback from other cortical regions, which acts to stabilize perception. Recent studies of bistable perception have emphasized the importance of top-down processing in resolving perceptual ambiguity and generating periods of stable percepts^5,6,33^. Specifically, multiple studies have used connectivity analyses, including dynamic causal modeling^34,35^ and granger causality^25^, to show that frontoparietal regions are likely vital for sending top-down signals during bistable perception. In relation to these results, alpha oscillations have been closely associated with top-down signaling in a variety of tasks and experiments^36–38^.

But how would top-down signals stabilize perception? One possibility is by carrying information about predictions made in frontoparietal regions. Under a predictive-coding model^26,39^, top-down predictions are compared with the input received in visual cortex, and then ultimately used to stabilize whichever percept is more likely at the moment based on a minimization of the prediction error. More broadly, classic perceptual inference models emphasize back-and-forth communication across the cortex involving top-down and bottom-up processes in order to resolve ambiguities inherent in natural vision^40,41^. These models focus on the use of Bayesian inference and present the possibility that top-down information may represent prior object knowledge and cues that can be used to stabilize a given percept.

Notably, alpha oscillations have been associated with a variety of functions other than top-down feedback^42^. In particular, the gating by inhibition model proposes that alpha oscillations reflect inhibition of activity in task-irrelevant brain regions^43,44^. Although the idea of inhibiting activity of specific neuronal populations is commonly implemented as a form of mutual inhibition in many computational models of bistable perception^45–50^, neural correlates of mutual inhibition among neuronal populations are difficult to measure experimentally. Furthermore, two previous findings give reason to believe the alpha effect we present here does not reflect the same mechanisms as mutual inhibition. First, Van Loon et al., 2013 administered lorazepam, a GABA_A_ receptor agonist, to subjects and found an increase in percept durations compared to placebo. The authors interpret this result as evidence for mutual inhibition acting to stabilize perception. Second, existing evidence shows that lorazepam actually decreases alpha power despite alpha’s well-established role in inhibiting cortical activity^51^. Therefore, if the alpha modulation we observed here reflects changes in mutual inhibition that can be manipulated by lorazepam, then alpha power should inversely correlate with percept duration—opposite to our experimental finding. In sum, we submit that a parsimonious explanation of our result is that alpha power fluctuations observed herein reflect the effect of top-down modulations in stabilizing perception.

### Possible functional roles of beta (13–30 Hz) oscillations

As with alpha, beta-band activity has also been linked with top-down signaling across a wide range of experiments^37,52,53^, see^54,55^ for reviews). However, the functional role of these top-down signals could very well be different from those reflected in alpha-band activity. In particular, our results fit well with a previous hypothesis^54^ suggesting that beta-band activity maintains the current cognitive or perceptual set by signaling the “status quo,” via interactions between higher-order cortical regions and sensory areas. Under this hypothesis, beta-band activity is higher when maintenance of a current percept, i.e. the “status quo,” is expected as opposed to when a change in perception is expected. Building on this hypothesis, Spitzer and Haegens^56^ propose a conceptual model for the functional role of beta oscillations, namely that beta-band activity can “(re)activate” latent neural ensembles that have previously encoded content-specific information. Under this model, during bistable perception, it is possible that neural ensembles can encode latent (i.e. without continuous spiking activity) representations of each percept of an ambiguous stimulus; the role of beta oscillations would then be to support “re(activation)” of the neural ensemble representing a percept, thus resulting in stronger perceptual memory.

Past experiments have also reported significant changes in beta-band activity around the times of perceptual switches^57–59^. Although these studies highlight the importance of beta oscillations during ambiguous perception, the exact role of beta activity remains unclear, with interpretations ranging from representing the likelihood of a percept being perceived^58^ to reflecting the “illusoriness” of a percept^59^. We extend on these findings by showing that the amplitude of beta oscillations correlates with percept duration, thus suggesting that beta activity is particularly important for stabilizing perception. Notably, these past experiments all found localized beta activity in parietal regions, where we also see the strongest effects in our results (Figs. 3C, 4B). In addition to these interpretations that offer possible explanations for the beta-band effect we observe, some important questions remain: are the functional roles of alpha and beta oscillations during bistable perception distinguishable, and what are their neural sources? Future investigation using intracranial recordings can better address these questions.

### Alpha and beta oscillations support perceptual memory

Although earlier work using intermittently presented ambiguous images mainly reported behavioral effects^18,19,60–62^ (see^22^ for a review), recent studies have begun to uncover neural underpinnings of the perceptual memory trace thought to be responsible for stabilizing perception. Using fMRI, Sterzer and Rees^63^ found a significant inter-individual correlation between perceptual stabilization and frontoparietal activation; Wang et al.^25^ reported that the content of perception and perceptual memory could be decoded from frontoparietal areas. In addition, functional connectivity during intermittent viewing is dominated by top-down influences across the cortex^25^. Together, these previous results suggest that frontoparietal regions and top-down signals play a key role in stabilizing perception when a stimulus is temporarily removed from view, possibly via a perceptual memory trace. In the context of oscillatory neural activity, the results we present here fall in line with these interpretations of top-down signaling: using two different analyses (Figs. 4, 5), we found converging evidence that alpha and beta activity supports perceptual memory. We interpret these results as reflecting top-down feedback that acts to stabilize perception. Notably, the association of alpha and beta activity with both perceptual stability during continuous viewing and perceptual memory during intermittent viewing suggests a unified set of neural mechanisms contributing to both phenomena.

The largest differences in amplitudes between stable and reversal trials were found during two time periods: (1) the transition from the end of the first image presentation into the blank period and (2) after the onset of the second image presentation (Fig. 4). The neural effects in these time periods may reflect different mechanisms: during the transition into the blank period, higher alpha and beta activity may reflect top-down signaling that promotes maintenance of the percept so that it is reinstated when the image reappears; on the other hand, higher alpha and beta amplitudes during the second image presentation may reflect reactivation of latent content. Interestingly, the relationship between alpha/beta amplitude and perceptual memory is weaker for the longest blank duration (1570 ms) (Figs. 4, 5), which may provide clues for how the duration of the blank period interacts with perceptual memory.

### Implications of null tDCS effect over rIFG

To our surprise, anodal tDCS over rIFG did not significantly influence perceptual switching behavior in either the continuous-viewing or the intermittent-viewing condition. Our choice of the rIFG for causal perturbation was motivated by a large body of previous work: (1) in the continuous viewing paradigm, numerous studies have shown that the rIFG activates around perceptual switching (reviewed in^6^) even when the reporting demand is removed^13^; (2) the rIFG encodes both the fluctuating perceptual content^25^ and prediction errors that slowly build up during a percept^26^; 3) in the intermittent-viewing paradigm, the rIFG encodes the content of perceptual memory and sends top-down influences to visual areas^25^. However, causal manipulation of the rIFG using tDCS was not carried out until now. We believe that our study conforms to a high standard: we used a double-blind and within-subject cross-over design. Therefore, any investigator bias or inter-subject variability could not have impacted the results. Moreover, experimental sessions conducted on different days showed high within-subject reliability (as measured by intraclass correlations), suggesting that behavioral measures are consistent within individuals and that day-to-day variance did not have a major influence on the results. Altogether, the null tDCS results we report here on behavior and neural activity are informative for future studies.

Interestingly, a recent TMS study using a structure-from-motion bistable stimulus^64^ reported that inhibitory (theta-burst) TMS of rIFG slowed down perceptual switching in the continuous-viewing condition. The discrepant results between this study and ours could be due to differences in experimental design (e.g., Weilnhammer et al.^64^ study was not double-blind), visual stimuli used, or stimulation techniques. The physiological bases of TMS and tDCS remain incompletely understood. Existing evidence suggests that inhibitory TMS depresses neural excitability potentially by inducing synaptic long-term depression; anodal tDCS enhances neural excitability by inducing tonic depolarization of resting membrane potential, resulting in increased spontaneous neuronal firing^65^ and synaptic long-term potentiation^66^. A limitation of the current study is that we were not able to confirm that tDCS had these effects on neural circuits due to the noninvasive nature of our electrophysiological recordings, thus it is possible that the null result we obtained was due to methodological issues such as not enough current reaching the rIFG or Occ during tDCS stimulation and/or the noninvasive EEG recordings not being able to measure tDCS-induced changes in the underlying neural circuits (which nonetheless did not have a behavioral effect).

### Conclusions and outlook

In conclusion, we report that alpha and beta oscillations contribute to promoting perceptual stability and perceptual memory when viewing ambiguous visual input. We suggest that these neural correlates reflect top-down feedback that acts to stabilize perception. We also report a null result that tDCS applied over rIFG did not influence perceptual behavior; however, further experiments will be necessary to ultimately determine whether rIFG plays a causal role in perceptual switching and/or stability. Future work can also help uncover specific functional roles of alpha and beta oscillations in perceptual stability. Doing so may have important implications for understanding abnormal neural processes in mental disorders such as autism spectrum disorder and bipolar disorder, both of which have been linked with unduly strong perceptual stability during bistable perception tasks^67–71^. More broadly, perceptual stability and perceptual memory are critical components of natural vision. In a world filled with ambiguous and fleeting visual signals, compounded by our frequent blinks and saccades, how is it that our visual experience appears seamless and stable? The neural correlates identified herein may provide clues for addressing this important question.

## Methods

### Subjects

The study was approved by the New York University School of Medicine Institutional Review Board under protocol s15-01323 (PI: Biyu He), and the experiment was conducted in accordance with NYUSoM IRB ethical guidelines. Thirty three right-handed healthy volunteers with normal or corrected-to-normal vision participated in the study. Data for twenty four subjects between 19 and 37 years old (mean age, 25.7; 15 females) were used for analyses in this paper. The other nine subjects were excluded from all analyses based on exclusion criteria during the initial screening session (details below in “Overall experimental design”). The study used a within-subject design, with twenty four subjects each undergoing three different HD-tDCS treatments, each on a different day. All subjects provided written informed consent in accordance with the Declaration of Helsinki.

### Overall experimental design

Twenty four subjects each participated in four experimental sessions. The purpose of the first session was to screen and familiarize the subject with the task. In the first session, subjects performed a shortened version of the task as practice (~ 30 min including both continuous and intermittent-viewing blocks). Data collected during this first session was not used in any analyses presented in this paper. The purpose of screening was to control for data quality. Subjects were excluded if one or more of the following exclusion criteria were met in the screening session: 1) Percentage of time with eyes closed (including blinks and saccades) while viewing images exceeded 5% (measured using Eyelink eye tracker); 2) Percentage of time spent in the “unsure” percept exceeded 20%; 3) Mean switches/min in the continuous viewing condition was either greater than 30 or less than 3. In total, nine subjects were excluded based on these criteria (six due to criterion #3; two due to criterion #2; one due to criterion #1) and did not participate in the main study described below.

The remaining twenty four subjects participated in three sessions involving different tDCS conditions (anodal-rIFG; anodal-Occ; sham). The following procedure was used in each of these sessions (Fig. 1A): (1) the subject completes the full-length task (~ 50 min) without EEG recording, (2) a Neuronavigation system (Rogue Research Inc.) was used to place the center tDCS electrode over the target brain region based on the subject’s own anatomical MRI (for details, see “Neurotargeting”), (3) deliver either sham or anodal tDCS to the target brain region, 4) mount the EEG cap and prepare electrodes for acceptable impedance (< 25 kOhm for active electrodes), 5) the subject completes the same task again under EEG recording. To summarize, during each experimental session, the subject performed the same task twice, once at the start of the session (pre-tDCS task without EEG) and once at the end of the session (post-tDCS task with EEG); in between, sham or anodal tDCS was applied to the target brain region. The post-tDCS task was completed within a window of 2 h after the end of tDCS stimulation. Due to the time required to prepare EEG electrodes, the post-tDCS task started on average 26.1 (s.d.: 6.0) minutes after the end of tDCS stimulation and was completed on average 79.5 (s.d.: 8.3) min after the end of tDCS stimulation. Different sessions were scheduled on separate days, with adjacent sessions spaced at least 3 days apart in order to avoid any after-effects of tDCS from the previous session. Previous studies showed that a single session of HD-tDCS, using parameters similar to the current design, has after-effects lasting at least 2 h, and no longer than 6 h^24^.

### Behavioral task

Two ambiguous images were used to study bistable perception: Rubin face-vase illusion and Necker cube. Both images were presented on a computer monitor (1920 × 1080 resolution, 60 Hz refresh rate). The face-vase image subtended 8.3° × 8.6° visual angle and was presented on a uniform gray background; the cube subtended 7.9° × 7.9° visual angle and was presented on a uniform white background. Two types of task conditions were performed by each subject: a continuous presentation condition and an intermittent presentation condition. The full-length task consisted of six blocks with the following condition sequence: continuous, intermittent, intermittent, continuous, intermittent, intermittent. In both block types, subjects were instructed to report their alternating percept by pressing two alternative buttons (percept-button mapping was counter-balanced across blocks); a third button corresponded to “unsure”. In both task conditions, subjects were instructed not to intentionally switch or hold percepts. The task was implemented using E-Prime software (Psychology Software Tools).

#### Continuous-viewing blocks

Each block consisted of six trials, with each trial consisting of: 2 s instruction screen, 2 s fixation screen, 60 s presentation of an ambiguous image, 2 s fixation screen (66 s total trial duration). The subject was instructed to fixate at the cross at the center of the screen throughout the task and indicate each time their percept changed. Each ambiguous image was presented in 3 trials within each block. The order of image presentation was pseudorandomized, and the total block time was 7.6 min.

#### Intermittent-viewing blocks

Each block consisted of six trials, with each trial consisting of: 2 s instruction screen, 2 s fixation screen, and then for 60 s there was a repeating sequence of 1.5 s presentation of an image followed by a blank screen (with only a fixation cross) of variable duration (64 s total trial duration). Within each trial, 1 of 3 possible durations was used for the blank period: 570 ms, 970 ms, or 1570 ms. These durations were chosen based on previous studies showing that varying the blank-period duration changes the perceptual reversal rate^17,19,20^; specifically, we were interested in using blank durations that resulted in gradually increased stabilization of the percept. The subject was instructed to fixate on the cross at the center of the screen throughout the task and indicate their percept each time the image appears on the screen and any time their percept switches while the image is on the screen. Each ambiguous image was presented in 3 trials within each block. Because there were three possible blank-period durations and two images, each block consisted of the same six types (2 images × 3 durations) of trials in a pseudorandomized order. The total block time was 7.4 min.

### Target brain regions

Two brain regions were targeted for stimulation: right inferior frontal gyrus (rIFG) and occipital pole (Occ). The locations of these brain regions were determined based on MNI coordinates published in past studies^6,25,27^ (rIFG: 54, 17, 14; Occ: 2, -99, 15). During each session, the subject received either sham or anodal tDCS over either rIFG or Occ. For all subjects, two of the three sessions were anodal stimulation over rIFG and Occ, respectively; in the third session, half of the subjects received sham stimulation over rIFG and the other half of the subjects received sham stimulation over Occ. The order of these 3 sessions was pseudorandomized and counterbalanced across all subjects. Data in the sham stimulation condition were pooled across rIFG sham and Occ sham sessions during analysis. For all sessions, both the subject and primary experimenter were blinded to whether sham or anodal stimulation was used (more information on blinding in “tDCS” section below).

### Neurotargeting

Anatomical magnetic resonance imaging (MRI) data were acquired for all subjects on a Siemens 7 T MRI scanner using a 32-channel head coil with internal head cage at New York University Center for Biomedical Imaging. High-resolution (1.0 mm isotropic voxels) T1-weighted MPRAGE images were acquired with the following parameters: FOV 256 mm, 192 sagittal slices, TR 3000 ms, TE 4.49 ms, flip angle 6°, fat suppression on. Stimulation sites were determined based on each subject’s anatomical MRI, using the Brainsight Neuronavigation system (Rogue Research Inc.). Each subject’s MRI data were registered to the standard MNI space using custom AFNI scripts for nonlinear transform. After the targeted coordinates were applied in MNI space, the MRI was transformed back into the individual subject’s head space for physical targeting. A mark was made on the skin at the scalp location nearest to the target region for placement of the tDCS anode. These methods closely follow those published in a previous study from our lab^23^.

### tDCS

tDCS current was generated by a neuroConn DC Stimulator Plus channeled through a 4 × 1 Multichannel Stimulation Device (Soterix Medical). A 4 × 1 montage of five sintered Ag/AgCl ring electrodes was used, consisting of one anode directly over the stimulation site surrounded by four equally spaced return electrodes at a radius of 5 cm from the anode. The electrodes were held in place in plastic electrode holders in a fitted cap (EASYCAP). The electrode holders were filled with SignaGel. Anodal stimulation consisted of a linear ramp-up period of 30 s at the beginning, followed by 20 min of sustained stimulation at 2.0 mA, and finally a linear ramp-down period of 30 s. Sham stimulation consisted of a 30 s ramp-up period to induce a tingling sensation, followed by 15 s of sustained stimulation at 2.0 mA, and finally a linear ramp-down period of 30 s; the subject then waited in the chair for 20 min to match the same duration as that of anodal stimulation^65^. During both anodal and sham tDCS, subjects were asked to relax and remain awake. In order to blind the main experimenter (M.Z.), the controls on the current generator used to start the anodal or sham stimulation were operated by another experimenter (R.H.) who was not significantly involved in data collection. The main experimenter (M.Z.) had no knowledge of tDCS condition during all data collection and initial analyses that do not require knowing the specific tDCS manipulation. Thus, this study adheres to the strictest double-blind procedure.

### EEG recording

Subjects were seated in a dimly lit, electromagnetic interference-shielded, soundproof room. 64 Ag/AgCl actiCAP EEG electrodes (Brain Products GmbH) were placed according to the international 10–10 system. Two reference electrodes were placed on the left and right mastoids. Four electrooculogram (EOG) electrodes were used: two above and below the left eye and two at the outer corner of each eye. Two electromyogram (EMG) electrodes were placed 4 cm apart over the right index finger flexor muscle (flexor digitorum superficialis), but EMG data were not used in analyses reported herein. All electrodes were prepared with 10% chloride ABRALYT HiCl abrasive electrolyte-gel (EASYCAP). EEG, EOG, and EMG data were collected using the BrainAmpDC system (Brain Products GmbH) in DC recording mode with a sampling rate of 1000 Hz. These methods closely follow those published in a previous study from our lab^23^.

### Eye tracking

An SR Research Eyelink 1000 + system was used to record the gaze position (x and y eye position) and pupil size of both eyes (1000 Hz) for all subjects while they performed the task. Subjects’ gaze and pupil sizes were determined by recording the pupil and corneal reflection of each eye. Pupil tracking was set to centroid model mode. A head-post with chin and forehead rests was used to stabilize the head while subjects performed the task.

### EEG preprocessing

EEG data were preprocessed using custom scripts in Matlab (MathWorks), EEGLAB^72^, and FieldTrip^73^. The EEG channels TP9 and TP10 were removed from all analyses unless otherwise noted because they were located over the mastoid reference electrodes and were thus not in contact with the skin. Exceptionally noisy channels were first removed by manual inspection and then interpolated based on the mean of neighboring channels (defined using a 10–10 system template). Continuous EEG and EOG data were bandpass filtered between 0.5 and 150 Hz and then notch filtered at 58–62 Hz. All filters were applied offline using a symmetrical Butterworth filter with zero phase shift. Independent component analysis (ICA) was then applied to all EEG and EOG electrodes; noisy components were removed, including blinking and eye movement artifacts. After removing ICA components, the data were low-pass filtered at 58 Hz. Lastly, a common average reference was applied to all channels.

### Behavioral measures

#### Continuous-viewing condition

Percept durations are defined as the duration from one button press until the next. Percept durations immediately before and after any “unsure” button presses were excluded. Because the durations preceding the first button press and after the last button press of each trial are uncertain (because they are bounded by the start and end of the trial, respectively), data for these durations were excluded from all analyses.

#### Intermittent-viewing condition

Stable blank periods are defined as blank periods for which the percept before and after the blank are the same and are not “unsure”. Reversal blank periods are those for which the percept before and after the blank are different and are not “unsure”. For each blank period, the percepts reported immediately before it and immediately after it were used for its definition (thus, within a 60 s trial, the number of reversals + number of stable + number of unsure equals the total number of blank periods). Raw reversal rate was calculated as the number of reversal blanks in a 60-s trial. To normalize for the different number of blank periods per unit time, we used the metric “probability of reversal,” which was calculated for each trial using the formula (number of reversals) / (number of stable + number of reversals). For all analyses using the intermittent-viewing data, one subject was excluded due to a technical error while running the intermittent-viewing task.

One subject was excluded from analyses using face-vase data due to the subject not reporting switches in any trials for at least one session (for both continuous-viewing and intermittent-viewing). Therefore, the total number of subjects entering into behavioral and neural analyses were as follows: Continuous viewing: Cube, N = 24; Face-vase, N = 23; Intermittent viewing: Cube, N = 23, Face-vase, N = 22.

Percentage changes from pre-tDCS to post-tDCS tasks were calculated using the formula: ((post-Tdcs − pre-tDCS)/pre-tDCS) × 100.

The violin plots for reporting behavioral results were created using a free toolbox^74^. Each plot includes a density estimate of the distribution (thick circle indicates mean and each thin circle represents one participant).

### Frequency-band-specific analyses

The data were filtered into 5 frequency bands using separate bandpass filters: 1–4 Hz (Delta), 4–8 Hz (Theta), 8–13 Hz (Alpha), 13–30 Hz (Beta), 30–55 Hz (Gamma). The amplitude envelope was extracted from the filtered signal in each frequency band, calculated by taking the absolute value of the Hilbert transform using Fieldtrip.

To test for differences in band-limited amplitude between tDCS conditions (Figs. 2B and S2B), we used the following procedure: (1) calculate the mean band-limited amplitude across each percept duration (for continuous-viewing trials) or across each blank period and its preceding image presentation (for intermittent-viewing trials); (2) calculate the mean across all “trial-means” calculated in step 1, resulting in a scalar value for band-limited amplitude at each channel for each subject; (3) perform a paired t-test between stimulation (rIFG or Occ) and sham at each channel across subjects. A similar analysis was applied to test for potential differences in band-limited amplitudes between tDCS conditions during eyes-closed rest recordings conducted immediately after finishing tDCS stimulation, which also revealed no significant differences (after correction for multiple comparisons across channels) between tDCS stimulation and sham sessions.

### Defining behavioral metric to index perceptual memory

For each 60-s intermittent-viewing trial, we segmented the trial into sets of consecutive stable blank intervals (Fig. 5A). A set of consecutive stable blanks was defined as the number of consecutive stable blanks before a switch was reported, therefore each set ended with a switch in percept after the last blank interval in the set. Each switch in percept that was preceded and followed by stable blank intervals were counted as “0” consecutive stable blanks (all sets of consecutive reversal blanks were excluded from this analysis). For example, if a subject reported the following sequence of percepts across 6 blank intervals, “face-face-face-vase-face-face-face,” this would be segmented into 2 sets of 2 consecutive stable blanks.

### Correlating band-limited amplitude with behavioral metrics

For the continuous-viewing data, for each electrode of each subject, we performed a Spearman rank correlation between percept duration and the mean band-limited amplitude over all percepts, pooled across all sessions. For the intermittent-viewing data, we performed a Spearman correlation between the perceptual memory metric and band-limited amplitude averaged across blank intervals over all “sets” (see previous section), pooled across all sessions. For both analyses, we calculated Fisher-z transformed Spearman rho values, which was subjected to a population level t-test. A cluster-based permutation tests^75^ was then conducted to correct for multiple comparisons across electrodes. This test consisted of the following steps: (1) uncorrected significant clusters of electrodes were determined based on channels with t-values that exceeded a threshold equivalent to *p* < 0.025 and were of the same sign; (2) the sum of the absolute values of t-values within each cluster was used as a summary statistic; (3) a null distribution of this summary statistic was generated by using 10,000 permutations of group labels and then calculating the largest summary statistic for each permutation; (4) each cluster determined in step 1 was compared to this null distribution, and those with a summary statistic exceeding the 97.5th percentile of the null distribution were considered significant (corresponding to *p* < 0.05 for a two-sided test).

## Supplementary Information

Supplementary Information.
