# Supplementary material for: Neural oscillations promoting perceptual stability and perceptual memory during bistable perception
Source: Sci Rep. 2022 Feb 17;12:2760. doi: 10.1038/s41598-022-06570-4 (PMC8854562; doi:10.1038/s41598-022-06570-4)
Supplement: Supplementary file 1 — Supplementary Information. [file 41598_2022_6570_MOESM1_ESM.docx]

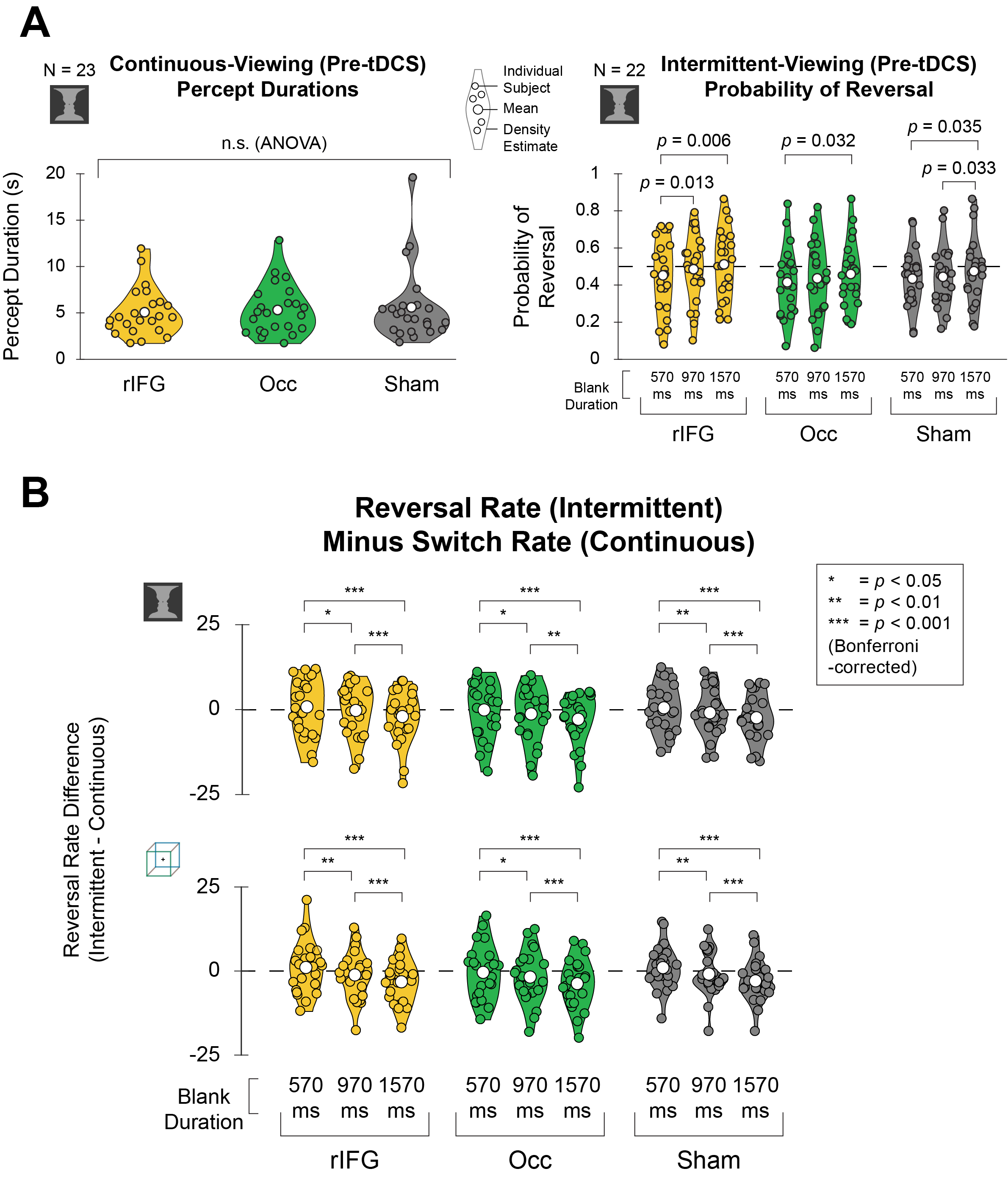


**Fig. S1. Pre-tDCS data (for face-vase trials) and reversal rates compared to continuous-viewing (for both images). (A)** Behavioral data across subjects for face-vase trials in the pre-tDCS task. Dotted line for intermittent-viewing data (*Right*) indicates 0.5 probability of reversal. **(B)** Raw reversal rates (i.e. number of switches/60 sec in intermittent-viewing condition) after subtracting switch rate in the continuous-viewing condition (calculated separately for each subject).


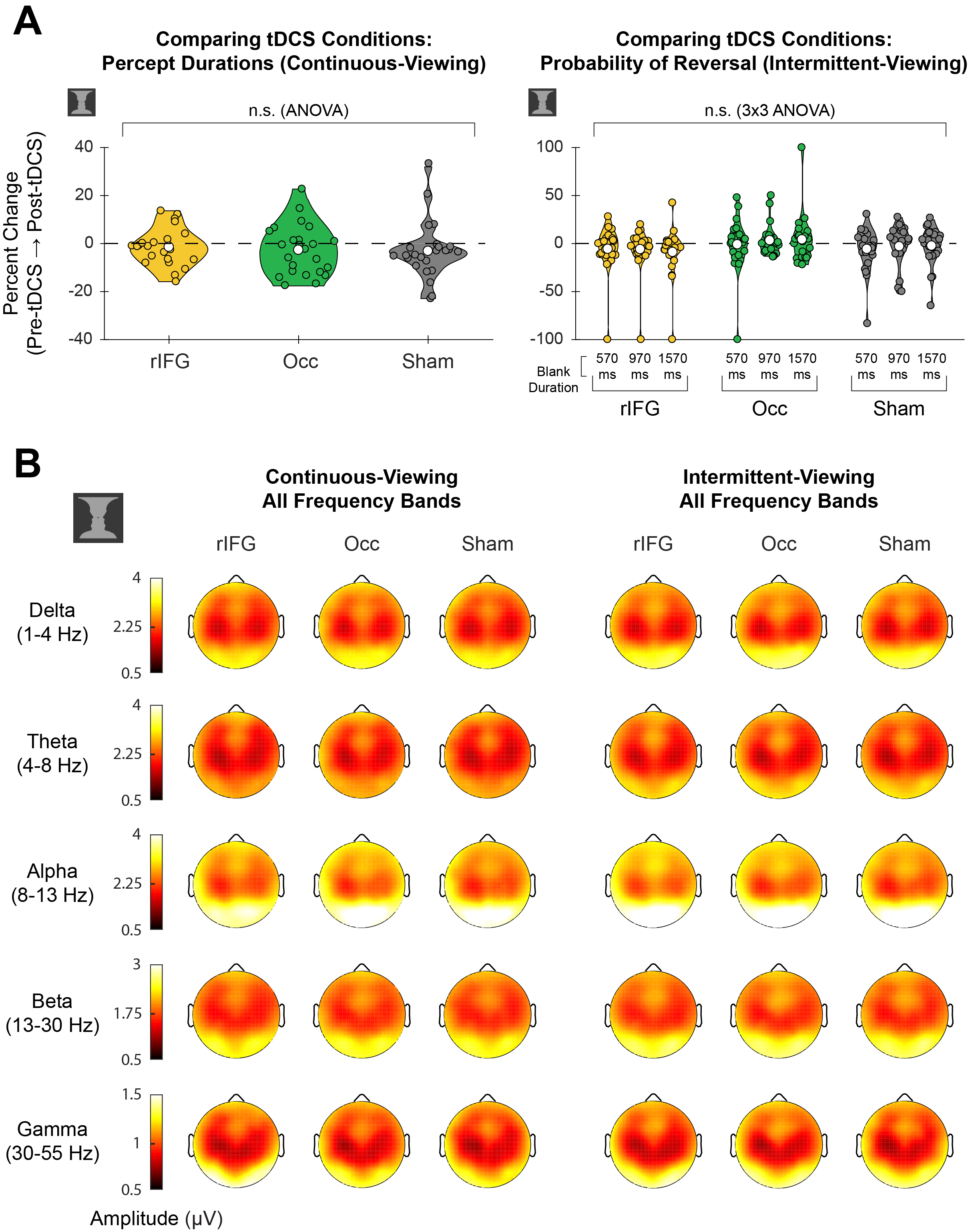


**Fig. S2. Face-vase trials: comparing tDCS conditions. (A)** Violin plots show %changes in behavioral measures from pre-tDCS task to post-tDCS task for face-vase trials. In the continuous-viewing condition (*Left*), percept durations did not differ significantly between tDCS conditions (repeated-measures ANOVA, F_2, 44_ = 1.062, *p* = 0.354). In the intermittent-viewing condition (*Right*), probability of reversal did not differ significantly between conditions (3x3 repeated-measures ANOVA, F_2, 42_ = 0.776, *p* = 0.467). **(B)** Topo-plots show mean amplitudes for each frequency band (*Rows*) during continuous-viewing (*Left*, *N* = 23) and in the 570-ms-blank trials of the intermittent-viewing condition (*Right*, *N* = 22) averaged across all subjects for face-vase trials. Amplitudes in each frequency band did not differ significantly between tDCS conditions (paired t-test, all *p* > 0.023, uncorrected across EEG channels).


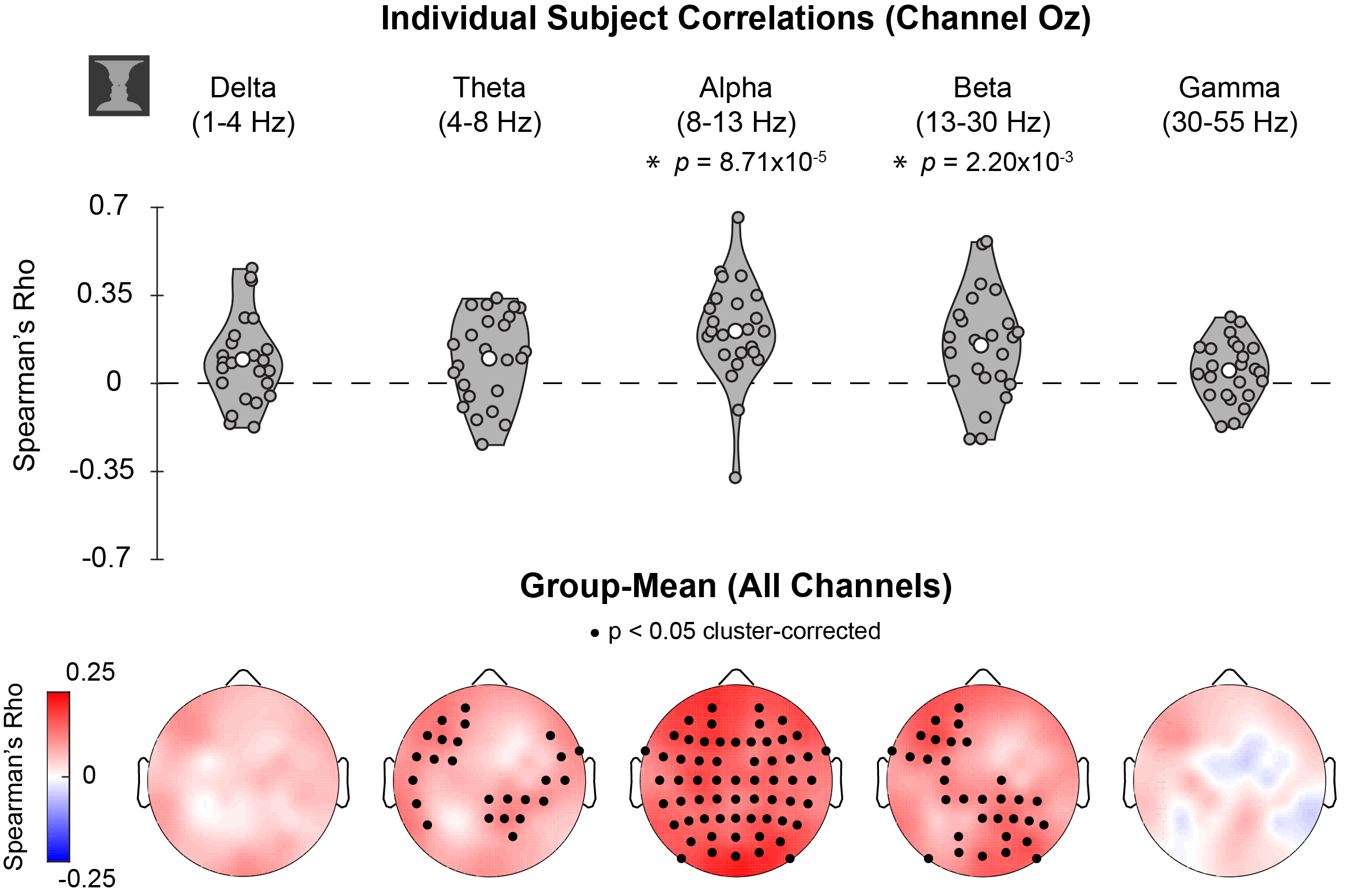


**Fig. S3. Face-vase trials: correlating percept durations and band-limited amplitudes.** Same as Fig. 3C but for face-vase trials.


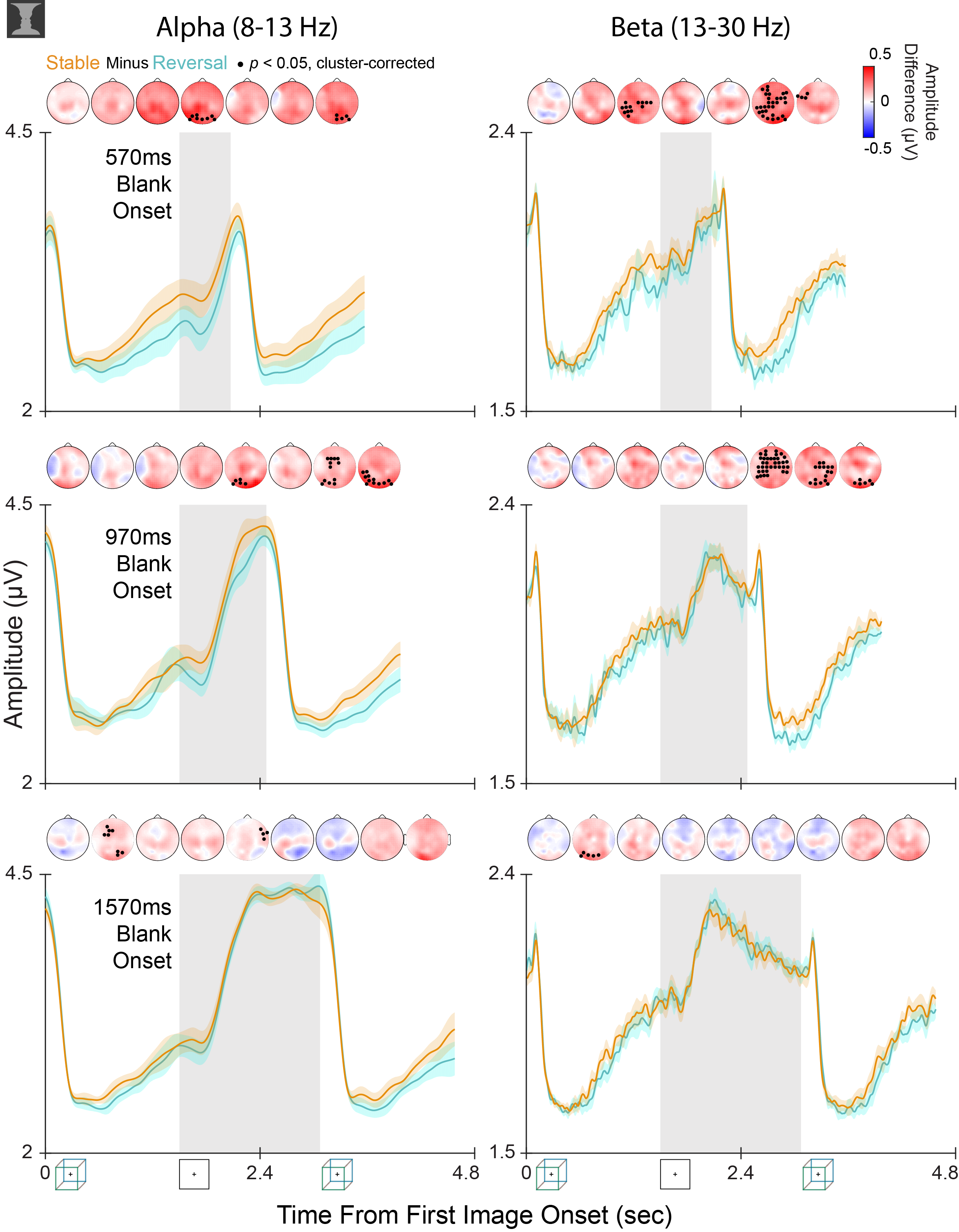


**Fig. S4. Face-vase trials: time courses for alpha and beta amplitudes in intermittent-viewing condition.** Same as Fig. 4B but for face-vase trials.


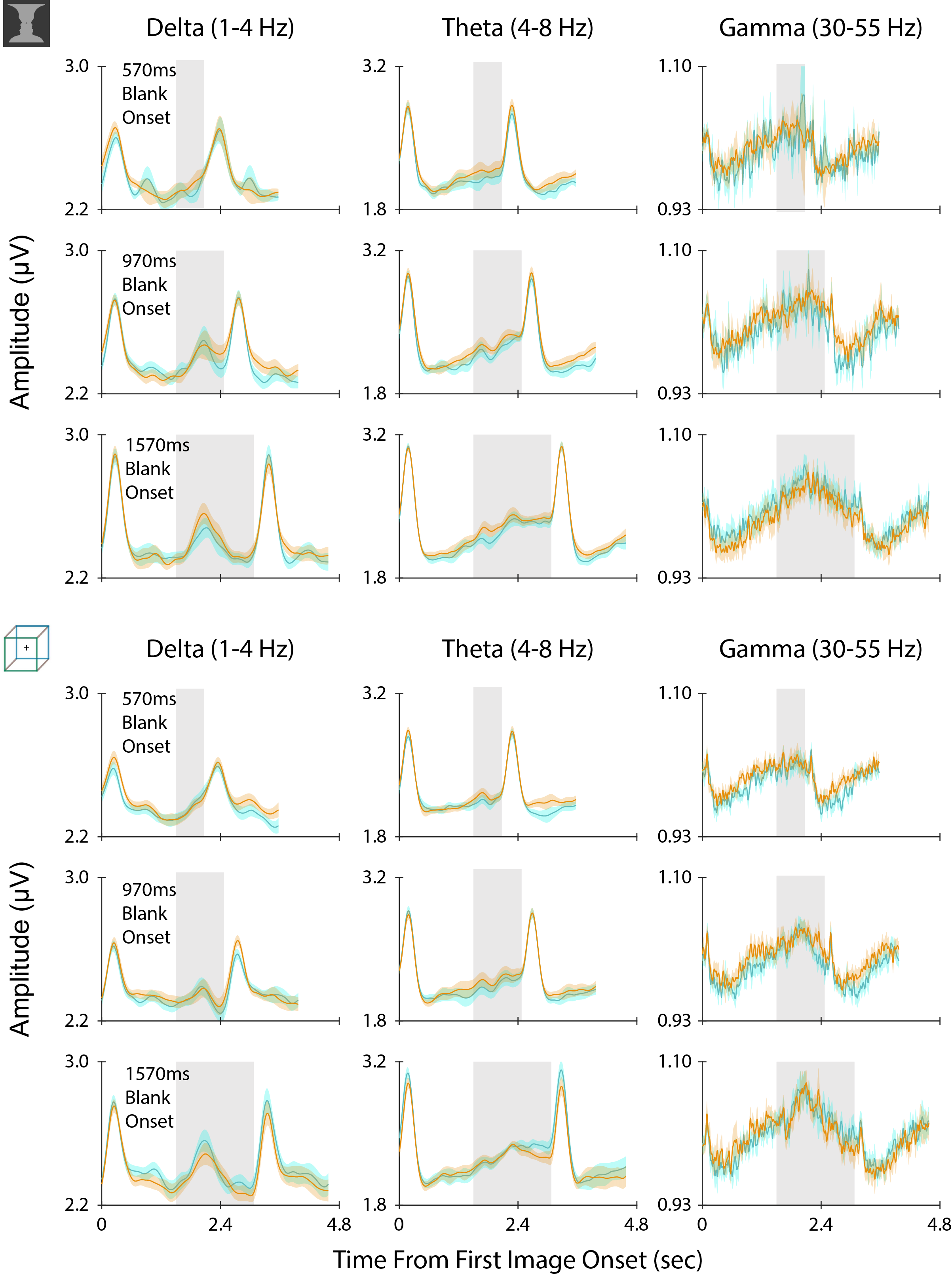
**Fig. S5. Time courses of amplitudes in all frequency bands in intermittent-viewing condition.** Same as curves in Fig. 4B and Fig. S4 but for additional frequency bands.


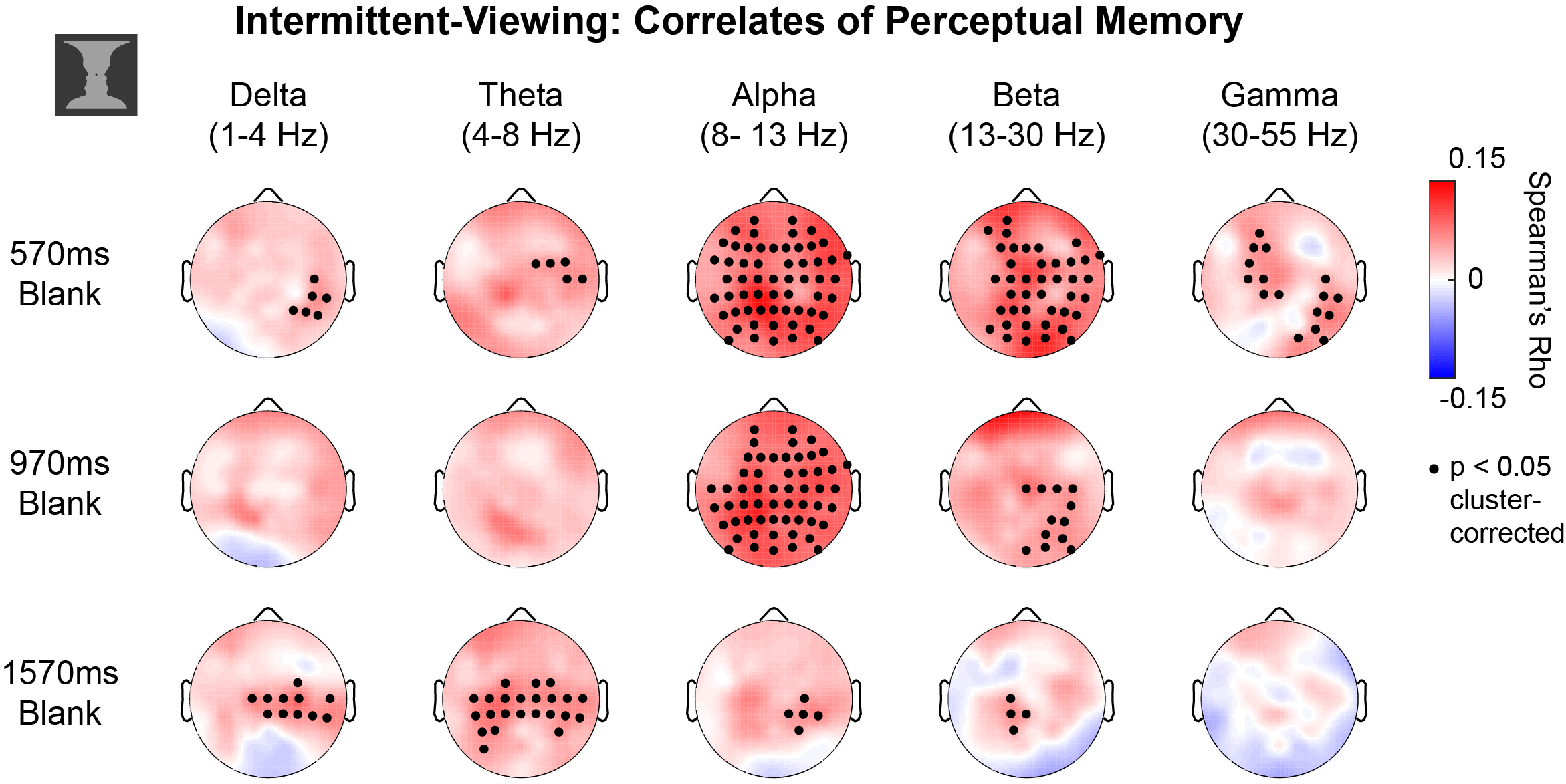


**Fig. S6. Face-vase trials: correlating persistence of perceptual memory and band-limited amplitudes.** Same as Fig. 5C but for face-vase trials.
